# The Effect of Educational Program on the Quality of Life and Self-Efficacy of the Mothers of the Infants with Congenital Heart Disease: A Randomized Controlled Trial

**Published:** 2014-01

**Authors:** Mitra Edraki, Mojgan Kamali, Noushin Beheshtipour, Hamid Amoozgar, Najaf Zare, Sedigheh Montaseri

**Affiliations:** 1 Community Based Psychiatric Care Research Center, Shiraz University of Medical Sciences, Shiraz, Iran;; 2 Department of Pediatric Nursing, College of Nursing and Midwifery, Shiraz University of Medical Sciences, Shiraz, Iran;; 3 Student Research Committee, Shiraz University Medical Sciences, Shiraz, Iran;; 4 Department of Pediatric Cardiology, Shiraz University of Medical Sciences, Shiraz, Iran; 5 Department of Biostatistics, School of Medicine, Shiraz University of Medical Sciences, Shiraz, Iran

**Keywords:** Congenital Heart Disease, Quality of Life, Self-Efficacy, Education, Mother

## Abstract

**Background:** Congenital heart disease causes large expenditures as well as mental pressures for the parents and, consequently, endangers the mothers’ quality of life and self efficacy. Thus, the present study aimed to determine the effect of educational program on the quality of life and self efficacy of the mothers of the infants with congenital heart disease.

**Methods:** The present randomized controlled trial was conducted on 56 mothers who had children with congenital heart disease (28 in the control and 28 in the intervention group) in Imam Reza Clinic, Shiraz, Iran in 2012. The mothers’ quality of life and self-efficacy were assessed using SF-36 and Sherer’s general self efficacy questionnaires before, immediately and 2 months after the training. The training was performed through four 90-minute sessions in 4 weeks. The data were analyzed using the SPSS statistical software and independent t-test, chi-square, and repeated measure analysis of variance.

**Results:** A significant differences was observed between the intervention and control groups regarding the mean of quality of life across the three study periods (F=59.91, P<0.0001). A significant difference was also found between the two groups concerning the mean of self efficacy at these times (F=114.11, P<0.0001).

**Conclusion:** According to the results, providing appropriate training for the mothers of the infants with congenital heart disease increased their quality of life as well as self-efficacy.

**Trial Registration Number: **IRCT2012080410489N1

## Introduction

Congenital Heart Disease (CHD) is a chronic disease,^1^ the second cause of death in infancy and childhood, and the only cause of heart disease in the children in developed countries.^2^ The prevalence of this disease has been reported differently in global statistics; however, it is quite important with the prevalence of 5-8 in 1000 live births. Up to now, more than 35 heart disorders have been identified with Ventricular Septal Defect (VSD) being the most prevalent one.^3^

Overall, diagnosis of CHD creates a lot of disappointment and anxiety for the parents; such a way that first they are shocked and then they feel great disappointment and anxiety related to the severity of the disease, type of medical procedures, and fear from death. The feeling of disappointment mostly results from the lack of information about the procedures and treatments, unfamiliarity with the 

hospital rules, unfriendliness of the staff, and fear from asking questions.^4^^,^^5^

In most of the communities, including Iran, mothers tolerate more mental pressure in taking care of the children compared to the fathers. In fact, they spend more time taking care of the children and are more responsible for making decisions about their treatment.^6^ In most of the cases, mothers seem to be trapped in a chain of caring for the children with no rest. Furthermore, taking care of the children with chronic diseases, such as CHD, has large expenditures for both the family and the society.^4^ These expenditures and mental pressures highly influence the parents’ quality of life.^3^^,^^5^ Kim et al. also believe that tension significantly affects the caregivers’ quality of life and changes four dimensions of psychological problems, mental function, physical function, and spiritual coping.^7^ Parents’ quality of life directly affects their children’s health.^8^ Based on the studies by Benzer et al., evaluation of life quality is one of the major predicators of health and improvement in chronic diseases because in such diseases, the goal of treatment is not only increasing the patients’ life time, but also improving their symptoms and performances.^9^

In general, quality of life is directly related to self-efficacy.^10^ Thus, both quality of life and self-efficacy are reduced in the mothers of the children with CHD. Self-efficacy is defined as the certainty by which, an individual successfully performs a particular behavior and expects the obtained results.^11^ Bandura believes that the parents’ self-efficacy is in fact their beliefs and capabilities in nurturing their children and is related to performance of both the family and the child.^12^ The parents’ low self efficacy leads to their tendency toward using negative ways of parenting and less utilization of treatment programs and services for their children. In order to feel self efficient, the parents need knowledge and information about the effective ways of taking care of the children.^13^ Education is a simple, inexpensive, and necessary instrument for the society’s health which eventually changes the behavior, creates a healthy life, and also plays a critical role in health and treatment fields.^14^ Training the patients and their families is one of the main components of care for the patients with chronic diseases and improvement of their health status. In fact, training creates a feeling of efficacy and reduces helplessness and anxiety. Consequently, it causes the patients to use more effective adaptability mechanisms and develop a more positive attitude. In this way, they will also be able to accept new roles, including caring for the children and provision of infants’ health.^4^^,^^15^

In addition, education is one of the duties of the health staff and one of the duties identified by the American Nurses Association.^16^ Considering what was mentioned above and the problems threatening the caregivers of the infants with CHD and their quality of life as well as self-efficacy, the present study aims to investigate the effect of educational program on the quality of life and self-efficacy of the mothers of the infants suffering from CHD.

## Materials and Methods

The present randomized controlled trial was conducted on the mothers of the infants with CHD in Imam Reza clinic, Shiraz, Iran in 2012. 

Based on d=15, α=0.05, δ=2o, β=0.20, and using the following formula, a 28-subject sample size was determined for the study (28 in each group):

n=[2(Z_1-(α/2)_+Z_(1-β)_)^ 2 ^σ^ 2 ^]/d^ 2^

**Figure 1:**
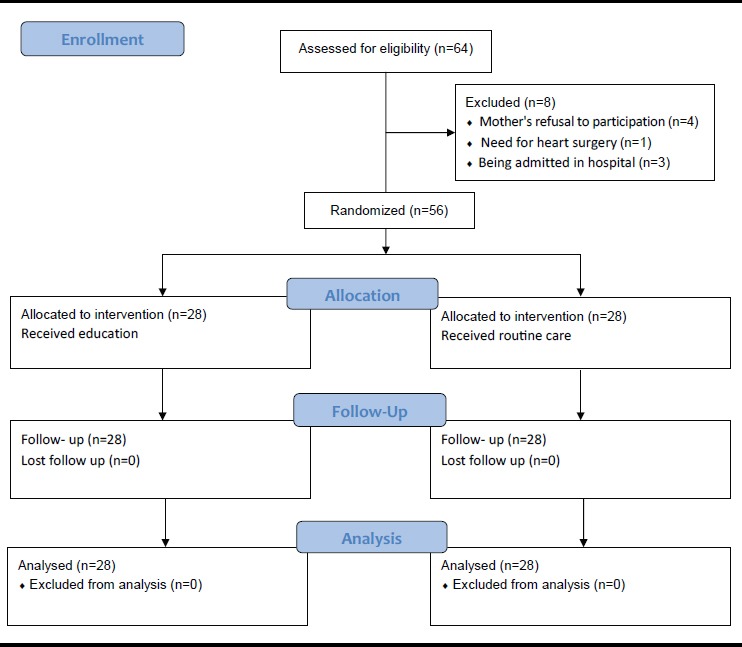
Diagram of the participants in the study

Overall, 64 mothers were assessed for eligibility. However, 8 mothers were excluded from the study due to their refusal to participation in the study (n=4), need for the heart surgery (n=1), and being admitted in the hospital. Finally, 56 mothers were entered into the study through convenience sampling. Then, they were randomly divided into an intervention and a control group through block randomization procedure with a random sequence of 4 block sizes. After sampling, the two groups were visited on different days in order to avoid information transfer between the study groups. It should be mentioned that none of the participants was excluded from the study during the follow up and data analysis. Figure 1 shows the diagram of the participants in this study.

The inclusion criteria of the study were the infant’s suffering from Atrial Septal Defect (ASD), VSD, and Patent Ductus Arteriosus (PDA), not having other chronic diseases, being below 12 months old, not having undergone heart surgery, existence of no other children with CHD in the family, passage of at least 2 weeks from diagnosis of CHD, and the mother’s being able to read and write. On the other hand, the exclusion criteria of the study were the infant’s death, the subjects’ unwillingness or impossibility to continue their cooperation, and the infant’s hospitalization during the intervention. 

The participants of the intervention group were divided into seven 4-subject groups and received the educational program through four 90-minute sessions in 4 weeks. This program was presented through power point and included information about the disease, types of the disease, causes, symptoms, diagnostic tests, treatment, its effect on the infant and the family, coping methods, taking care of such infants at home, nutrition, preventing infection, vaccination, and medication. The intervention group subjects were emphasized not to explain the educational program and give any information to the other subjects. The participants of the control group only received the follow-up care without any educational programs. After data collection, the booklet of educational program was given the control group, as well.

The outcomes of this study were quality of life and self–efficacy. The data were collected at the beginning of the study and at the end of the 8^th^ week.

The study data were collected using three questionnaires.

*Demographic Information Questionnaire*

This questionnaire included mother’s characteristics, such as age, level of education, occupation, and consanguineous marriage, as well as infant’s information, including age, sex, and mode of delivery.

*Generalized Self Efficacy (GSE) Scale*

Generalized Self Efficacy (GSE) scale was designed by Sherer and Madox in 1982 and consists of 23 items. Among these 23 items, 17 ones are related to general self-efficacy, while the remaining 6 ones are related to the experiences of self-efficacy in social situations. In the present study, the researchers made use of the 17-item GSE scale which includes 17 five-point items ranging from completely agree to completely disagree. In this questionnaire, a point from 1 to 5 is allocated to each item. Of course, the scores of the items 1, 3, 8, 9, 13, and 15 are increased from left to right, while those of the remaining items are increased from right to left. Overall, the minimum and maximum scores are 17 and 85, respectively.^17^

Sherer et al. (1982) reported a Cronbach’s alpha of 0.86 for this questionnaire. In addition, Beirami (2006) conducted a study entitled “The effect of teaching the social skills on the social efficacy of the students” and confirmed the internal consistency of the scale by Cronbach’s alpha of 0.79.^18^ Najafi et al. conducted a study entitled “The relationship between self-efficacy and mental health in high school students” and reported the reliability of the questionnaire to be 0.80. In addition, the concurrent validity of the questionnaire by concurrent administration of symptom checklist-90 revised was 0.45.^19^

In this study, the reliability coefficient (Cronbach’s α) for the total score of GSE was 0.76.

*Quality of Life Questionnaire (SF-36)*

Quality of life questionnaire (SF-36) was designed by Var and Sherbon in the U.S. in 1992.^20^ It was translated into Persian by Montazeri et al. in 2005 and its reliability and validity were investigated. The questionnaire revealed to have both appropriate reliability (Cronbach’s alpha=0.7) and convergent validity (correlation coefficient=0.4). Narimani (2008) investigated the effect of education on the hemodialysis patients’ quality of life and reported Cronbach’s alpha of 0.68 for the questionnaire.^20^

The quality of life questionnaire aims to evaluate both physical and mental health of the individuals and consists of 36 items assessing 8 health domains; i.e., general health, physical performance, limitation in role performance due to physical reasons, limitation in role performance due to emotional reasons, physical pain, mental health, fatigue or exhilaration, and social function. The minimum and maximum scores of this questionnaire are 0 and 100, respectively. The questionnaire included yes/no, 3-option (scored as 0, 50, and 100), 5-option (scored as 0, 25, 50, 75, and 100), and 6-option items (scored as 0, 20, 40, 60, 80, and 100). Except for yes/no items, the responses to the subscales were interpreted as a mean from 0 to 100.^21^

In this study, the reliability coefficient (cronbachs α) for the total score of the quality of life questionnaire was 0.91. 

This study was approved by the Ethics Committee of Shiraz University of Medical Sciences. Besides, written informed consents were obtained from all the subjects. It was also explained to the subjects that participation in this study was voluntary and participation / non participation did not affect their follow-up care.

The study data were analyzed using the SPSS statistical software (v. 15) and independent t-test, paired *t* test, Chi-square, and repeated measurement test. In addition, P<0.05 was considered as statistically significant.

## Results

Overall, 56 mothers participated in the current study. The mean±SD age of the mothers was 28.3±5.6 and 28±5.3 years in the intervention and the control group, respectively. In addition, the mean age of the infants in the intervention and the control group was 6±3.3 and 6.2±3.5 months, respectively. Socio-demographic characteristics of the mothers and their infants are show in table 1. The study results revealed no statistically significant difference between the two groups regarding the socio-demographic characteristics (table 1).

**Table 1:** Characteristics of the study mothers and their infants

**Variables**	**Intervention group**		**Control group**			**P value**
*Mother’s age*						
mean±SD	28.3±5.6		28.0±5.3			P=0.848
*Infant’s age*						
mean±SD	6.0±3.3		6.2±3.5			P=0.846
*Infant’s sex; N (%)*						
Girl	14 (50.0)				17 (60.7)	P=0.420
Boy	14 (50.0)				11 (39.3)	
*Mother’s occupation; N (%)*						
Homemaker	27 (96.4)			26 (92.9)		P=0.5
Employed	1 (3.6)			2 (7.1)		
*Consanguineous Marriage; N (%)*						
Yes	14 (50.0)				19 (67.9)	P=0.174
No	14 (50.0)				9 (32.1)	
*Mode of delivery; N (%)*						
Natural	11 (39.3)				13 (46.4)	P=0.589
Caesarean section	17 (60.7)				15 (53.6)	
*Level of education; N (%)*						
Primary school		7 (25.0)			6 (21.4)	P=0.953
Middle school		6 (21.4)			5 (17.9)	
High school& diploma		11 (39.3)			13 (46.4)	
Above diploma		4 (14.3)			4 (14.3)	

According to table 2, no significant difference was found between the study groups regarding the quality of life before the study (P=0.91). However, a significant difference was observed between the intervention and control groups regarding the mean of quality of life immediately and 2 months after the intervention (F=59.91, P=0.001)

**Table 2:** Comparison of the two groups regarding the mean of quality of life before, immediately, and 2 months after the intervention

***Quality of life***	**Before the intervention**		**Immediately** **After the intervention**	**2 months** **After the intervention**		**Repeated Measures Analysis of Variance**			
	**mean±SD**		**mean±SD**	**mean±SD**		**Between Groups**			**Within-Subjects**
*Total Quality of Life*									
Intervention	44.5±17.6		68.0±12.6	62.8±13.0		F=14.09, <0.0001	F=59.91, <0.0001
Control	45±16.2		44.5±15.7	44.1±13.6			
*Domains of Quality of Life *									
*Physical performance*									
Intervention		68.0±26.3	82.1±18.4		79.4±19.7		F=.88, 0.35	F=14.57, <0.0001
Control		69.8±26.8	71.2±23.9		71.2±25.4			
*Limitation in role performance due to physical reasons*									
Intervention		45.5±34.0	60.7±26.7		58.0±28.9		F=.66, 0.41	F=3.61, 0.06
Control		47.3±31.4	50.0±34.0		48.4±34.8			
*Fatigue and exhilaration*									
intervention		36.6±24.3	55.7±19.0		48.5±19.0		F=9.72, 0.003	F=18.36, <0.0001
Control		31.0±20.6	31.4±19.8		30.5±18.1			
*Mental health*									
Intervention		36.8±25.9	61.5±18.8		57.7±19.6		F=10.58, 0.002	F=16.77, <0.0001
Control		34.0±20.7	35.8±20.6		36.8±18.1			
*Social function*									
Intervention		41.3±28.9	76.7±20.8		71.8±18.5		F=11.22, 0.001	F=55.00, <0.0001
Control		45.5±28.9	41.5±29.0		39.7±26.3			
*Pain*									
Intervention		55.0±28.3	65.9±23.3		66.0±22.2		F=0.08, 0.77	F=20.54, <0.0001
Control		65.1±28.7	57.9±26.1		58.3±24.3			
*General health*									
Intervention		40.5±17.2	67.5±17.7		61.2±16.1		F=12.94, 0.001	F=48.68, <0.0001
Control		38.7±19.4	43.0±14.2		41.7±16.6			
*Limitation in role performance due to emotional reasons*									
Intervention		32.1±35.6	73.8±24.6		59.5±29.1		F=16.68, <0.0001	F=18.85, <0.0001
Control		28.5±32.3	25.0±30.9		26.1±31.8			

At the beginning of the study, no significant difference was indicated between the two groups concerning the mean of self-efficacy. However, repeated measures analysis of variances demonstrated a significant difference between the intervention and the control group regarding the mean of self-efficacy immediately and two months after the intervention (F=114.11, P<0.0001) (table 3).

**Table 3:** Comparison of the two groups regarding the mean of self-efficacy before and immediately and 2 months after the intervention

***Self-efficacy***	**Before the intervention**	**Immediately** **after the intervention**	**2 months** **after the intervention**	**Repeated Measures Analysis of Variance**	
	**mean±SD**	**mean±SD**	**mean±SD**	**Between groups**	**Within-Subjects**
Intervention	37.7±6.8	57.1±10.3	52.4±8.4	F=10.12	F=114.11
Control	39.7±8.3	39.6±8.8	40.6±8.5	P<0.0001	P<0.0001

## Discussion

The results of the present study showed that the educational intervention improved the life quality of the mothers of the infants suffering from CHD. This finding is in line with that of the study conducted by in Babolsar, Iran in order to investigate the effect of training how to take care of the children with cerebral palsy on the life quality of their caregivers. The results of that study showed a 20-point increase in the caregivers’ life quality scores after the training.^22^ In another study, in order to determine the effect of progressive muscle relaxation program on the life quality and self-efficacy of multiple sclerosis patients in Shahr-e-kord, Iran also, a significant increase was observed in the life quality scores of the intervention group caregivers after the training (P<0.001).^16^ One other study was performed in Germany to assess the effect of rehabilitation program on the life quality of the parents of the children suffering from chronic diseases (cardiovascular diseases and cancer). In that study, the parents’ quality of life improved immediately and 6 months after the intervention.^23^ In the same line, Grey et al. reported a significant improvement in the parents’ quality of life after teaching the adaptability skills to the parents of the children with type I diabetes.^24^ Safe and inexpensive programs for empowering the mothers should be considered as a priority. Overall, it can be implied that the educational intervention was more effective in improvement of life quality compared to the informal and unorganized trainings provided for the control group.

The findings of the present study also confirmed the effect of the educational program on the self-efficacy of the mothers of the infants with CHD, which is in agreement with the results of another study showing the effectiveness of the educational program in increasing the self-efficacy of the parents of the children suffering from Asperger syndrome.^25^ In the same line, after execution of supportive and educational programs for the parents of the children with disabilities in Barlo’s study, a considerable increase was observed in the parents’ self-efficacy scores (P<0.004).^26^ On the other hand, in the study conducted by Sarabi et al. (2010) in Mashhad in order to investigate the effect of training the parents on self-efficacy of the mothers of the children with autism, no significant increase was found in the mothers’ self-efficacy after the training. This might be due to the emotional problems as well as lack of social support for such parents in Iran.^27^ In a study on the effect of training on the parents of the children suffering from asthma, the parents’ quality of life and self-efficacy significantly improved after the intervention.^13^ Training the parents increases their knowledge and improves their performance. This can subsequently have caused changes in the parental performance and self-efficacy.

Overall, considering the findings of the current research and those of the previous studies, one can claim that education can improve the life quality and self-efficacy of the parents of the children suffering from chronic diseases. Lazarous and Folkman believed that a large number of individuals adapted to stress through searching for information and improving their cognitive skills.^28^ Furthermore, it is stated that high complications of chronic diseases required trained, professional caregivers at the time of discharge and at home.^29^

One of the limitations of the current study was the parents’ lack of cooperation or discontinuation of taking part in the study during the intervention due to the lack of knowledge and transportation problems. Of course, when they were informed about the advantages of participating in the study and provided with the transportation costs, this problem was solved to some extent. One other study limitation was the possibility to obtain information about the disease, care, and treatment from the treatment team; i.e., physicians or nurses, in the two groups.

After all, future studies are needed to be conducted on the effect of education on the life quality and self-efficacy of the children suffering from CHD as well as the parents of the children with other chronic diseases.

## Conclusion

According to the results, providing appropriate training for the mothers of the infants with CHD increased their quality of life as well as self-efficacy. Since nurses play a critical role in educational affaires of counseling programs and interventions, they can improve the parents’ quality of life through increasing their knowledge and information. 
